# Taxonomic relationship between *Amolopsminutus* Orlov & Ho, 2007 and *A.ottorum* Pham, Sung, Pham, Le, Zieger & Nguyen, 2019, with first record of *A.minutus* from China (Anura, Ranidae)

**DOI:** 10.3897/BDJ.12.e129214

**Published:** 2024-09-26

**Authors:** Shuo Liu, Truong Quang Nguyen, Minh Duc Le, Cuong The Pham, Anh Van Pham, Mian Hou, Hongxin Zhou, Mingzhong Mo, Mei Li, Biao Li, Xiong Luo, Dingqi Rao, Song Li

**Affiliations:** 1 Kunming Natural History Museum of Zoology, Kunming Institute of Zoology, Chinese Academy of Sciences, Kunming, China Kunming Natural History Museum of Zoology, Kunming Institute of Zoology, Chinese Academy of Sciences Kunming China; 2 Institute of Ecology and Biological Resources, Vietnam Academy of Science and Technology, Hanoi, Vietnam Institute of Ecology and Biological Resources, Vietnam Academy of Science and Technology Hanoi Vietnam; 3 Graduate University of Science and Technology, Vietnam Academy of Science and Technology, Hanoi, Vietnam Graduate University of Science and Technology, Vietnam Academy of Science and Technology Hanoi Vietnam; 4 Faculty of Environmental Sciences, University of Science, Vietnam National University, Hanoi, Vietnam Faculty of Environmental Sciences, University of Science, Vietnam National University Hanoi Vietnam; 5 Central Institute for Natural Resources and Environmental Studies, Vietnam National University, Hanoi, Vietnam Central Institute for Natural Resources and Environmental Studies, Vietnam National University Hanoi Vietnam; 6 Department of Herpetology, American Museum of Natural History, New York, United States of America Department of Herpetology, American Museum of Natural History New York United States of America; 7 College of Continuing (Online) Education, Sichuan Normal University, Chengdu, China College of Continuing (Online) Education, Sichuan Normal University Chengdu China; 8 Kunming Institute of Zoology, Chinese Academy of Sciences, Kunming, China Kunming Institute of Zoology, Chinese Academy of Sciences Kunming China; 9 Honghe Prefecture Forestry and Grassland Bureau of Yunnan Province, Mengzi, China Honghe Prefecture Forestry and Grassland Bureau of Yunnan Province Mengzi China; 10 Guanyinshan Provincial Nature Reserve Management and Protection Bureau, Yuanyang, China Guanyinshan Provincial Nature Reserve Management and Protection Bureau Yuanyang China; 11 Yunnan Key Laboratory of Biodiversity Information, Kunming Institute of Zoology, Chinese Academy of Sciences, Kunming, China Yunnan Key Laboratory of Biodiversity Information, Kunming Institute of Zoology, Chinese Academy of Sciences Kunming China

**Keywords:** 16S, Guanyinshan Provincial Nature Reserve, morphology, synonym, taxonomy, torrent frogs

## Abstract

**Background:**

Based on the examination of specimens of *Amolopsminutus* Orlov & Ho, 2007 and *A.ottorum* Pham, Sung, Pham, Le, Zieger & Nguyen, 2019, we found that there is no significant morphological difference between them. Phylogenetic analysis also showed that *A.minutus* and *A.ottorum* belong to the same taxon. In addition, we discovered the distribution of *A.minutus* in China.

**New information:**

In this study, we provide the first molecular data of *Amolopsminutus* and regard *A.ottorum* as a junior synonym of *A.minutus*. In addition, we report the first record of *A.minutus* from China, based on nine specimens collected from Guanyinshan Provincial Nature Reserve in southern Yunnan Province and present an updated diagnosis of this species, based on literature data and newly-collected specimens.

## Introduction

The torrent frogs of *Amolops* Cope, 1865 inhabits torrents or waterfalls and they usually have an abdominal sucker in larvae and enlarged digital discs in adults (Fei et al. 2009, Fei et al. 2012). This genus is distributed broadly from southern and eastern Himalayas to Peninsular Malaysia (Dever et al. 2012, Pham et al. 2019, Wu et al. 2020, Mahony et al. 2022, Tang et al. 2023, Li et al. 2024, Frost 2024). It currently contains 86 recognised species (Frost 2024) and they are placed into ten species groups, namely the *A.chayuensis* group, *A.daiyunensis* group, *A.hainanensis* group, *A.larutensis* group, *A.mantzorum* group, *A.marmoratus* group, *A.monticola* group, *A.ricketti* group, *A.spinapectoralis* group and *A.viridimaculatus* group (Jiang et al. 2021, Wu et al. 2024).

The *Amolopsmantzorum* group currently comprises twelve species, namely *A.ailao* Tang, Sun, Liu, Luo, Yu & Du, 2023, *A.dafangensis* Li, Liu, Ke, Cheng & Wang, 2024, *A.granulosus* (Liu & Hu, 1961), *A.jinjiangensis* Su, Yang & Li, 1986, *A.lifanensis* (Liu, 1945), *A.loloensis* (Liu, 1950), *A.mantzorum* (David, 1872), *A.minutus* Orlov & Ho, 2007, *A.ottorum* Pham, Sung, Pham, Le, Zieger & Nguyen, 2019, *A.sangzhiensis* Qian, Xiang, Jiang, Yang & Gui, 2023, *A.shuichengnicus* Lyu & Wang, 2019 and *A.tuberodepressus* Liu & Yang, 2000 (Tang et al. 2023, Li et al. 2024).

*Amolopsminutus* was described by Orlov and Ho (2007), based on morphological characteristics from Tam Duong District, Lai Chau Province, Vietnam, and molecular data of the species is unavailable. *Amolopsottorum* was discovered by Pham et al. (2019), based on two female specimens from Muong La District, Son La Province, Vietnam. The type localities of both species are located in north-western Vietnam and they are approximately 90 km distant from each other.

During our fieldwork in northern Vietnam, we collected fifteen specimens of *Amolopsminutus* from its type locality in Lai Chau Province. As these specimens morphologically resemble *A.ottorum*, we suspected that these two species may be conspecific. To confirm their taxonomic status, we sequenced the homologous gene of the topotypic specimens of *A.minutus* and the type specimens of *A.ottorum* and phylogenetic analysis supported that they are conspecific with a genetic divergence of 0.7% (16S gene). Morphological examination of the type specimens of *A.minutus* showed that the original description of the species was not very accurate and there was no significant morphological difference between *A.minutus* and *A.ottorum*. According to the order of naming time, we herein consider *A.ottorum* as a junior synonym of *A.minutus*. In addition, we recently collected nine specimens of *Amolops* from Guanyinshan Provincial Nature Reserve in Yunnan Province, China. The sequences of these specimens clustered with those of the topotypic specimens of *A.minutus*. We herein report the record of *A.minutus* from China for the first time.

## Materials and methods

Field surveys were conducted in Lai Chau Province, northern Vietnam, in 2020 and in Guanyinshan Provincial Nature Reserve, Yunnan Province, China, in 2023 and 2024. Voucher specimens collected from Vietnam were deposited at the Institute of Ecology and Biological Resources (IEBR), Hanoi, Vietnam and specimens collected from China were deposited at Kunming Natural History Museum of Zoology, Kunming Institute of Zoology, Chinese Academy of Sciences (KIZ), Kunming, China.

Measurements were taken with a digital caliper to the nearest 0.1 mm. The following morphological characteristics were used: snout-vent length (SVL), from the tip of the snout to the cloacal; head length (HL), from the rear of the lower jaw to the tip of the snout; head width (HW), at the greatest cranial width; snout-eye distance (ESL), from the tip of the snout to the anterior corner of the eye; eye diameter (ED), horizontal diameter of the eye; tympanum diameter (TD), horizontal diameter of the tympanum; fore-limb length (FLL), from the tip of the disc of the third finger to the axilla; hind-limb length (HLL), from the tip of the disc of the fourth toe to the groin; tibia length (TL), from the knee to the tarsus; foot and tarsus length (FOT), from the tip of the disc of the fourth toe to the posterior edge of the tibia.

Total genomic DNA was extracted from tissues. A fragment of the 16S ribosomal RNA (16S) gene was amplified and sequenced. The primer pairs L2188: 5’–AAAGTGGGCCTAAAAGCAGCCA–3’ and 16H1: 5’–CTCCGGTCTGAACTCAGATCACGTAGG–3’ (Hedges 1994, Matsui et al. 2006) and 16SAR: 5’-CGCCTGTTTAYCAAAAACAT-3’ and 16SBR: 5’-CCGGTYTGAACTCAGATCAYGT-3’ (Simon et al. 1994) were used in polymerase chain and cycling reactions. All newly-generated sequences have been deposited in GenBank and homologous sequences of other *Amolops* species were obtained from GenBank (Table 1).

Sequences were aligned using MAFFT 7.471 (Katoh and Standley 2013). The best fit substitution models were selected for Bayesian inference (BI) and maximum likelihood (ML) analyses, respectively, using the corrected Akaike Information Criterion (AICc) in ModelFinder (Kalyaanamoorthy et al. 2017). Bayesian inference was performed in MrBayes 3.2.7 (Ronquist et al. 2012) using the GTR+F+I+G4 model. Markov chains were run for 5,000,000 generations and sampled every 100 generations. The first 25% of the sampled trees was discarded as burn-in and the remaining trees were used to estimate Bayesian posterior probabilities (BPPs). Maximum likelihood analysis was performed in IQ-TREE 1.6.12 (Nguyen et al. 2015) with the TIM2+F+I+G4 model. Branch support was assessed using 5,000 ultrafast bootstrap replicates (UFB). The values of BPPs and UFB ≥ 95% are considered strong support for a clade (Ronquist et al. 2012, Nguyen et al. 2015). The uncorrected pairwise distances between species were calculated in MEGA 11 (Tamura et al. 2021).

## Taxon treatments

### Amolops minutus

Orlov & Ho, 2007

605428C5-FBCE-5ABC-9C40-3708D5821590

#### Materials

- **Type status:** Other material. **Occurrence:** catalogNumber: IEBR A.5142; individualCount: 1; sex: male; lifeStage: adult; occurrenceID: D85CF383-2685-58BF-B761-02FA97632765; **Taxon:** scientificName: Amolopsminutus; **Location:** country: Vietnam; stateProvince: Lai Chau; locality: Ho Thau Village, Ho Thau Commune, Tam Duong District; verbatimElevation: 2440 m; verbatimCoordinates: 22°24′42″N 103°36′35″E; **Event:** eventRemarks: collected by Anh Van Pham, Chung Van Hoang, Tien Phan Quang, and Nenh Ba Sung in May 2020; **Record Level:** basisOfRecord: preserved specimen
- **Type status:** Other material. **Occurrence:** catalogNumber: IEBR A.5143; individualCount: 1; sex: male; lifeStage: adult; occurrenceID: 220E4937-E84B-5FA0-B0DE-88D8039C4940; **Taxon:** scientificName: Amolopsminutus; **Location:** country: Vietnam; stateProvince: Lai Chau; locality: Ho Thau Village, Ho Thau Commune, Tam Duong District; verbatimElevation: 2440 m; verbatimCoordinates: 22°24′42″N 103°36′35″E; **Event:** eventRemarks: collected by Anh Van Pham, Chung Van Hoang, Tien Phan Quang, and Nenh Ba Sung in May 2020; **Record Level:** basisOfRecord: preserved specimen
- **Type status:** Other material. **Occurrence:** catalogNumber: IEBR A.5144; individualCount: 1; sex: male; lifeStage: adult; occurrenceID: B1C10D49-59B9-5180-9D18-54C1BC04E812; **Taxon:** scientificName: Amolopsminutus; **Location:** country: Vietnam; stateProvince: Lai Chau; locality: Ho Thau Village, Ho Thau Commune, Tam Duong District; verbatimElevation: 2440 m; verbatimCoordinates: 22°24′42″N 103°36′35″E; **Event:** eventRemarks: collected by Anh Van Pham, Chung Van Hoang, Tien Phan Quang, and Nenh Ba Sung in May 2020; **Record Level:** basisOfRecord: preserved specimen
- **Type status:** Other material. **Occurrence:** catalogNumber: IEBR A.5145; individualCount: 1; sex: male; lifeStage: adult; occurrenceID: 24C4943D-F3CA-5BF0-B133-87C5CB7A1603; **Taxon:** scientificName: Amolopsminutus; **Location:** country: Vietnam; stateProvince: Lai Chau; locality: Ho Thau Village, Ho Thau Commune, Tam Duong District; verbatimElevation: 2440 m; verbatimCoordinates: 22°24′42″N 103°36′35″E; **Event:** eventRemarks: collected by Anh Van Pham, Chung Van Hoang, Tien Phan Quang, and Nenh Ba Sung in May 2020; **Record Level:** basisOfRecord: preserved specimen
- **Type status:** Other material. **Occurrence:** catalogNumber: IEBR A.5146; individualCount: 1; sex: male; lifeStage: adult; occurrenceID: 5CEB76ED-5893-519D-9452-E3D31153B175; **Taxon:** scientificName: Amolopsminutus; **Location:** country: Vietnam; stateProvince: Lai Chau; locality: Ho Thau Village, Ho Thau Commune, Tam Duong District; verbatimElevation: 2440 m; verbatimCoordinates: 22°24′42″N 103°36′35″E; **Event:** eventRemarks: collected by Anh Van Pham, Chung Van Hoang, Tien Phan Quang, and Nenh Ba Sung in May 2020; **Record Level:** basisOfRecord: preserved specimen
- **Type status:** Other material. **Occurrence:** catalogNumber: IEBR A.5147; individualCount: 1; sex: male; lifeStage: adult; occurrenceID: C23C4993-DBF6-512A-BA1F-007CFABBA6BA; **Taxon:** scientificName: Amolopsminutus; **Location:** country: Vietnam; stateProvince: Lai Chau; locality: Ho Thau Village, Ho Thau Commune, Tam Duong District; verbatimElevation: 2440 m; verbatimCoordinates: 22°24′42″N 103°36′35″E; **Event:** eventRemarks: collected by Anh Van Pham, Chung Van Hoang, Tien Phan Quang, and Nenh Ba Sung in May 2020; **Record Level:** basisOfRecord: preserved specimen
- **Type status:** Other material. **Occurrence:** catalogNumber: IEBR A.5148; individualCount: 1; sex: male; lifeStage: adult; occurrenceID: 9319A5D0-DB68-5859-BD38-3E0623489A2C; **Taxon:** scientificName: Amolopsminutus; **Location:** country: Vietnam; stateProvince: Lai Chau; locality: Ho Thau Village, Ho Thau Commune, Tam Duong District; verbatimElevation: 2440 m; verbatimCoordinates: 22°24′42″N 103°36′35″E; **Event:** eventRemarks: collected by Anh Van Pham, Chung Van Hoang, Tien Phan Quang, and Nenh Ba Sung in May 2020; **Record Level:** basisOfRecord: preserved specimen
- **Type status:** Other material. **Occurrence:** catalogNumber: IEBR A.5149; individualCount: 1; sex: male; lifeStage: adult; occurrenceID: FD7307D9-575F-559F-B23E-5A7DBC70CC7B; **Taxon:** scientificName: Amolopsminutus; **Location:** country: Vietnam; stateProvince: Lai Chau; locality: Ho Thau Village, Ho Thau Commune, Tam Duong District; verbatimElevation: 2440 m; verbatimCoordinates: 22°24′42″N 103°36′35″E; **Event:** eventRemarks: collected by Anh Van Pham, Chung Van Hoang, Tien Phan Quang, and Nenh Ba Sung in May 2020; **Record Level:** basisOfRecord: preserved specimen
- **Type status:** Other material. **Occurrence:** catalogNumber: IEBR A.5150; individualCount: 1; sex: female; lifeStage: adult; occurrenceID: 0F19C455-DBF5-5A49-9C5C-B047533698E6; **Taxon:** scientificName: Amolopsminutus; **Location:** country: Vietnam; stateProvince: Lai Chau; locality: Ho Thau Village, Ho Thau Commune, Tam Duong District; verbatimElevation: 2440 m; verbatimCoordinates: 22°24′42″N 103°36′35″E; **Event:** eventRemarks: collected by Anh Van Pham, Chung Van Hoang, Tien Phan Quang, and Nenh Ba Sung in May 2020; **Record Level:** basisOfRecord: preserved specimen
- **Type status:** Other material. **Occurrence:** catalogNumber: IEBR A.6299; individualCount: 1; sex: female; lifeStage: adult; occurrenceID: 14D431C9-4554-500C-BD95-364B33E742E2; **Taxon:** scientificName: Amolopsminutus; **Location:** country: Vietnam; stateProvince: Lai Chau; locality: Ho Thau Village, Ho Thau Commune, Tam Duong District; verbatimElevation: 2440 m; verbatimCoordinates: 22°24′42″N 103°36′35″E; **Event:** eventRemarks: collected by Anh Van Pham, Chung Van Hoang, Tien Phan Quang, and Nenh Ba Sung in May 2020; **Record Level:** basisOfRecord: preserved specimen
- **Type status:** Other material. **Occurrence:** catalogNumber: IEBR A.6300; individualCount: 1; sex: female; lifeStage: adult; occurrenceID: AF82375D-18FF-54D1-B211-8AC61B87CF47; **Taxon:** scientificName: Amolopsminutus; **Location:** country: Vietnam; stateProvince: Lai Chau; locality: Ho Thau Village, Ho Thau Commune, Tam Duong District; verbatimElevation: 2440 m; verbatimCoordinates: 22°24′42″N 103°36′35″E; **Event:** eventRemarks: collected by Anh Van Pham, Chung Van Hoang, Tien Phan Quang, and Nenh Ba Sung in May 2020; **Record Level:** basisOfRecord: preserved specimen
- **Type status:** Other material. **Occurrence:** catalogNumber: IEBR A.6301; individualCount: 1; sex: female; lifeStage: adult; occurrenceID: F4EB3970-70D7-5F67-8EFA-6A5734985252; **Taxon:** scientificName: Amolopsminutus; **Location:** country: Vietnam; stateProvince: Lai Chau; locality: Ho Thau Village, Ho Thau Commune, Tam Duong District; verbatimElevation: 2440 m; verbatimCoordinates: 22°24′42″N 103°36′35″E; **Event:** eventRemarks: collected by Anh Van Pham, Chung Van Hoang, Tien Phan Quang, and Nenh Ba Sung in May 2020; **Record Level:** basisOfRecord: preserved specimen
- **Type status:** Other material. **Occurrence:** catalogNumber: IEBR A.6302; individualCount: 1; sex: female; lifeStage: adult; occurrenceID: B11DEB88-8877-5909-AC5D-806099C697B0; **Taxon:** scientificName: Amolopsminutus; **Location:** country: Vietnam; stateProvince: Lai Chau; locality: Ho Thau Village, Ho Thau Commune, Tam Duong District; verbatimElevation: 2440 m; verbatimCoordinates: 22°24′42″N 103°36′35″E; **Event:** eventRemarks: collected by Anh Van Pham, Chung Van Hoang, Tien Phan Quang, and Nenh Ba Sung in May 2020; **Record Level:** basisOfRecord: preserved specimen
- **Type status:** Other material. **Occurrence:** catalogNumber: IEBR A.6303; individualCount: 1; sex: female; lifeStage: adult; occurrenceID: 7B75B4E5-270B-5227-9928-67B20CB682FB; **Taxon:** scientificName: Amolopsminutus; **Location:** country: Vietnam; stateProvince: Lai Chau; locality: Ho Thau Village, Ho Thau Commune, Tam Duong District; verbatimElevation: 2440 m; verbatimCoordinates: 22°24′42″N 103°36′35″E; **Event:** eventRemarks: collected by Anh Van Pham, Chung Van Hoang, Tien Phan Quang, and Nenh Ba Sung in May 2020; **Record Level:** basisOfRecord: preserved specimen
- **Type status:** Other material. **Occurrence:** catalogNumber: IEBR A.6304; individualCount: 1; sex: female; lifeStage: adult; occurrenceID: B7189F6D-EFF6-5110-A7B6-DE4DBB8C2E2E; **Taxon:** scientificName: Amolopsminutus; **Location:** country: Vietnam; stateProvince: Lai Chau; locality: Ho Thau Village, Ho Thau Commune, Tam Duong District; verbatimElevation: 2440 m; verbatimCoordinates: 22°24′42″N 103°36′35″E; **Event:** eventRemarks: collected by Anh Van Pham, Chung Van Hoang, Tien Phan Quang, and Nenh Ba Sung in May 2020; **Record Level:** basisOfRecord: preserved specimen
- **Type status:** Other material. **Occurrence:** catalogNumber: KIZ 2023064; individualCount: 1; sex: male; lifeStage: adult; occurrenceID: 518014E8-3FCE-56F9-B84A-BC2D935425CA; **Taxon:** scientificName: Amolopsminutus; **Location:** country: China; stateProvince: Yunnan; locality: Ganiang Township, Yuanyang County, Honghe Prefecture; verbatimElevation: 2410 m; verbatimCoordinates: 23°0'57"N 102°56'57"E; **Event:** eventRemarks: collected by Shuo Liu on 16 May 2023; **Record Level:** basisOfRecord: preserved specimen
- **Type status:** Other material. **Occurrence:** catalogNumber: KIZ 2023065; individualCount: 1; sex: male; lifeStage: adult; occurrenceID: 0FC83528-7760-5F20-84F7-3B4465EEECBD; **Taxon:** scientificName: Amolopsminutus; **Location:** country: China; stateProvince: Yunnan; locality: Ganiang Township, Yuanyang County, Honghe Prefecture; verbatimElevation: 2410 m; verbatimCoordinates: 23°0'57"N 102°56'57"E; **Event:** eventRemarks: collected by Shuo Liu on 16 May 2023; **Record Level:** basisOfRecord: preserved specimen
- **Type status:** Other material. **Occurrence:** catalogNumber: KIZ 2023066; individualCount: 1; sex: male; lifeStage: adult; occurrenceID: 89044FE6-F2FA-5991-9BC8-A22EBEFFDD45; **Taxon:** scientificName: Amolopsminutus; **Location:** country: China; stateProvince: Yunnan; locality: Ganiang Township, Yuanyang County, Honghe Prefecture; verbatimElevation: 2410 m; verbatimCoordinates: 23°0'57"N 102°56'57"E; **Event:** eventRemarks: collected by Shuo Liu on 16 May 2023; **Record Level:** basisOfRecord: preserved specimen
- **Type status:** Other material. **Occurrence:** catalogNumber: KIZ 2023067; individualCount: 1; sex: male; lifeStage: adult; occurrenceID: DFC7F447-5B74-5FFD-9254-C729C75CC841; **Taxon:** scientificName: Amolopsminutus; **Location:** country: China; stateProvince: Yunnan; locality: Ganiang Township, Yuanyang County, Honghe Prefecture; verbatimElevation: 2410 m; verbatimCoordinates: 23°0'57"N 102°56'57"E; **Event:** eventRemarks: collected by Shuo Liu on 16 May 2023; **Record Level:** basisOfRecord: preserved specimen
- **Type status:** Other material. **Occurrence:** catalogNumber: KIZ 2023068; individualCount: 1; sex: male; lifeStage: adult; occurrenceID: DC4AB2B4-70F8-516E-A561-24B34E657C26; **Taxon:** scientificName: Amolopsminutus; **Location:** country: China; stateProvince: Yunnan; locality: Ganiang Township, Yuanyang County, Honghe Prefecture; verbatimElevation: 2410 m; verbatimCoordinates: 23°0'57"N 102°56'57"E; **Event:** eventRemarks: collected by Shuo Liu on 16 May 2023; **Record Level:** basisOfRecord: preserved specimen
- **Type status:** Other material. **Occurrence:** catalogNumber: KIZ 2023069; individualCount: 1; sex: female; lifeStage: adult; occurrenceID: 2467D2C6-A15C-5EE0-8C7E-B63F9F489211; **Taxon:** scientificName: Amolopsminutus; **Location:** country: China; stateProvince: Yunnan; locality: Ganiang Township, Yuanyang County, Honghe Prefecture; verbatimElevation: 2410 m; verbatimCoordinates: 23°0'57"N 102°56'57"E; **Event:** eventRemarks: collected by Shuo Liu on 16 May 2023; **Record Level:** basisOfRecord: preserved specimen
- **Type status:** Other material. **Occurrence:** catalogNumber: KIZ 2023070; individualCount: 1; sex: female; lifeStage: adult; occurrenceID: FDAC765F-29ED-5487-9B51-46F6331679CF; **Taxon:** scientificName: Amolopsminutus; **Location:** country: China; stateProvince: Yunnan; locality: Ganiang Township, Yuanyang County, Honghe Prefecture; verbatimElevation: 2410 m; verbatimCoordinates: 23°0'57"N 102°56'57"E; **Event:** eventRemarks: collected by Shuo Liu on 16 May 2023; **Record Level:** basisOfRecord: preserved specimen
- **Type status:** Other material. **Occurrence:** catalogNumber: KIZ 2023102; individualCount: 1; sex: female; lifeStage: adult; occurrenceID: A891C6DE-2573-5D59-A021-CE1C3EA24FD3; **Taxon:** scientificName: Amolopsminutus; **Location:** country: China; stateProvince: Yunnan; locality: Xiaoxinjie Township, Yuanyang County, Honghe Prefecture; verbatimElevation: 2490 m; verbatimCoordinates: 22°59'20"N 102°59'26"E; **Event:** eventRemarks: collected by Shuo Liu on 20 May 2023; **Record Level:** basisOfRecord: preserved specimen
- **Type status:** Other material. **Occurrence:** catalogNumber: KIZ 2024112; individualCount: 1; sex: male; lifeStage: adult; occurrenceID: F47A3080-9562-550A-B16F-1828B9EA9F16; **Taxon:** scientificName: Amolopsminutus; **Location:** country: China; stateProvince: Yunnan; locality: Ganiang Township, Yuanyang County, Honghe Prefecture; verbatimElevation: 2200 m; verbatimCoordinates: 23°2'54"N 102°54'45"E; **Event:** eventRemarks: collected by Shuo Liu on 13 April 2024; **Record Level:** basisOfRecord: preserved specimen

#### Extended diagnosis

This species is assigned to the *Amolopsmantzorum* species group on the basis of the absence of a dorsolateral fold and the absence of circum-marginal groove on the disc of the first finger. It is distinguishable from other members of this species group by a combination of the following morphological characters: size small (SVL 29.7–38.3 mm in adult males and 38.5–51.1 mm in adult females); head moderate large, longer than wide (HL/SVL 0.33–0.37 in males and 0.31–0.35 in females, HW/SVL 0.28–0.33 in males and 0.26–0.32 in females); tympanum distinct, small (TD/HL 0.10–0.23 in males and 0.09–0.27 in females); pineal spot present; pupil oval, horizontal; vomerine teeth absent or weakly developed; vocal sac absent in males; fore-limbs robust (FLL/SVL 0.70–0.87 in males and 0.67–0.85 in females), relative finger lengths I < II < IV < III; hind-limbs long (HLL/SVL 1.79–2.09 in males and 1.70–2.00 in females), relative toe length I < II < III < V < IV; dorsal skin smooth, with a few flattened tubercles on flanks and posterior surface of dorsum; supratympanic fold absent or weakly developed; true dorsolateral folds absent, but dorsolateral glandular folds distinct; circummarginal groove on tip of first finger absent; inner metatarsal tubercle small; outer metatarsal tubercle absent; nuptial pad present on finger I of adult males. Colour of dorsal surface from nearly uniform green to mostly brown, flanks mostly green, dark bars on dorsal limbs distinct or indistinct.

#### Distribution

**Figure 1.**
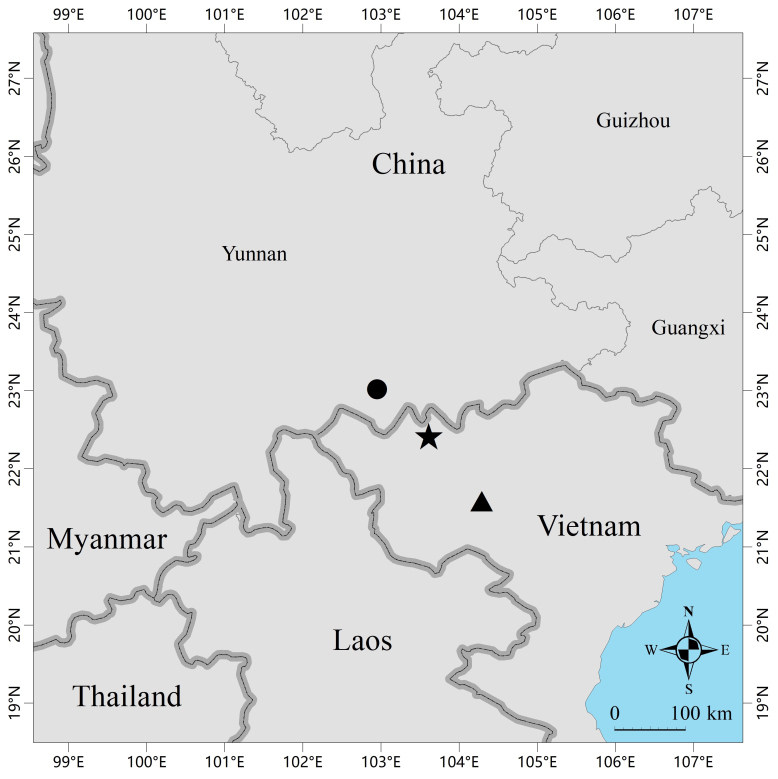
Map showing the type locality (black star) of *Amolopsminutus* in Lai Chau Province, Vietnam, the type locality (black triangle) of *Amolopsottorum* in Son La Province, Vietnam and the collection site (black dot) of the specimens from Guanyinshan Provincial Nature Reserve in Yuanyang County, Honghe Prefecture, Yunnan Province, China.

*Amolopsminutus* is currently known from Lai Chau and Son La provinces in northern Vietnam and Guanyinshan Provincial Nature Reserve in Yuanyang County, Honghe Prefecture, Yunnan Province, China (Fig. 1).

## Analysis

**Figure 2.**
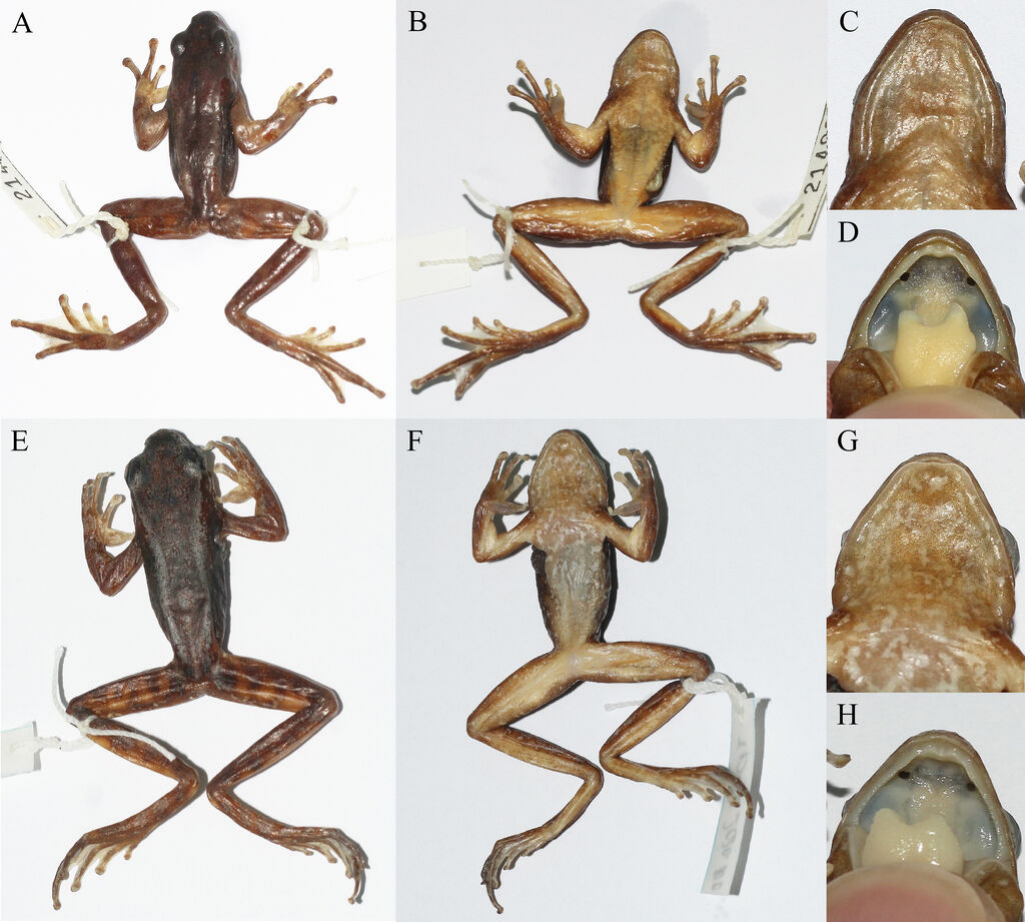
Dorsal view (A), ventral view (B), close-up view the gular region (C) and close-up view of the upper oral wall (D) of the paratype (ZISP 7615) of *Amolopsminutus*; and dorsal view (E), ventral view (F), close-up view the gular region (G) and close-up view of the upper oral wall (H) of the topotypic specimen (IEBR A.5142) of *A.minutus*.

Morphological characteristics of the specimens collected from the type locality of *Amolopsminutus* in Lai Chau Province (Vietnam) were similar to those in the original description of Orlov and Ho (2007), except for the vomerine teeth which were absent or weakly developed and the gular pouches being absent in males (vs. vomerine teeth strongly developed and paired gular pouches present in males in the original description) and small variations in the relative size of the tympanum and the relative length of the fore-limbs (Table 2). However, we found that the type specimens of *A.minutus* have no vomerine teeth or only weakly-developed vomerine teeth and no gular pouches in males (Fig. 2), which were not as described in Orlov and Ho (2007).

Moreover, morphological comparison showed that the type specimens of *A.ottorum* from Son La Province (Vietnam) and the newly-collected specimens of *Amolops* from Yunnan Province (China) are very similar to *A.minutus* (Table 2).

**Figure 3.**
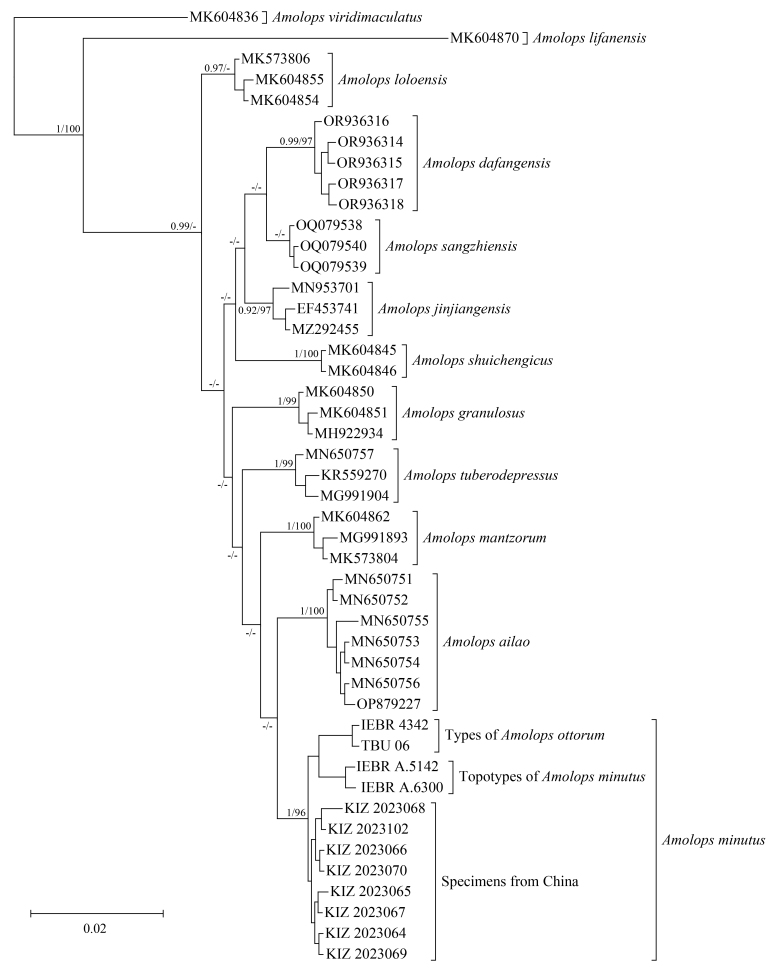
Bayesian phylogram of the *Amolopsmantzorum* group inferred from the 16S rRNA sequences. Numbers after and behind “/” are BPPs and UFB values (only above 0.90/90 are shown), respectively.

The newly-generated sequences are approximately 850 bp or 570 bp. BI and ML analyses yielded similar results. The sequences of the type specimens of *Amolopsottorum* from Son La Province (Vietnam), the specimens collected from the type locality of *A.minutus* in Lai Chau Province (Vietnam) and the *Amolops* specimens collected from Yunnan Province (China), clustered in the same clade with strong support by both BI and ML (BPPs = 1, UFB = 96) (Fig. 3). The uncorrected pairwise distance between the sequences of the type specimens of *A.ottorum* and the specimens collected from the type locality of *A.minutus* was 0.7% and the uncorrected pairwise distance between the sequences of the *Amolops* specimens collected from Yunnan and the specimens collected from the type locality of *A.minutus* was 0.6% (Table 3).

Based on morphological data and phylogenetic analysis, we assign the specimens collected from the type locality of *A.minutus* in Lai Chau Province (Vietnam) and from Yunnan Province (China) to *A.minutus* and regard *A.ottorum* as a junior synonym of *A.minutus*.

## Discussion

**Figure 4.**
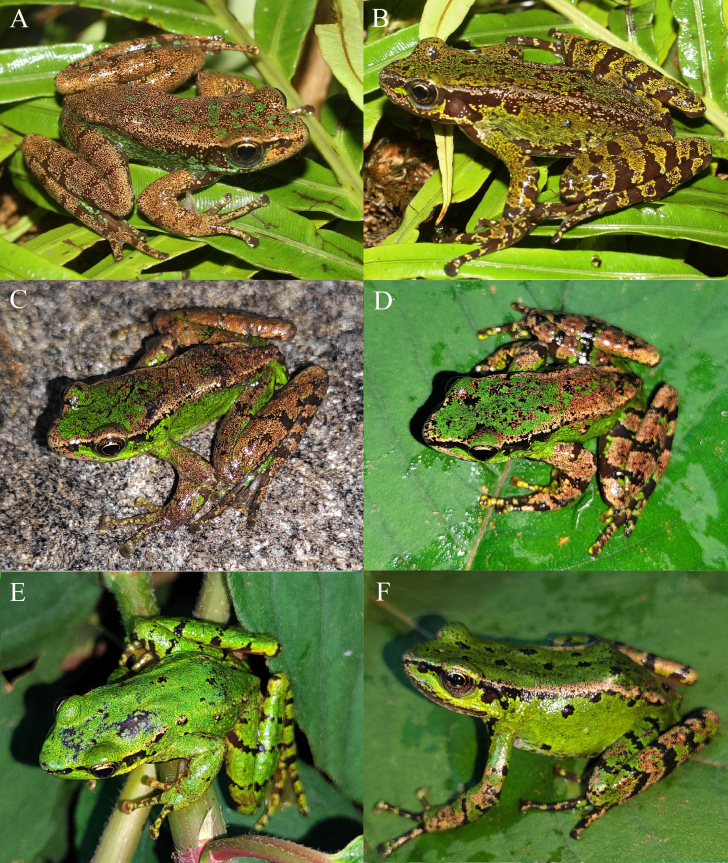
The specimens of *Amolopsminutus* in life. A, the male topotypic specimen (IEBR A.5142); B, the female topotypic specimen (IEBR A.6300); C, the male specimen (KIZ 2024112) from China; D, the male specimen (KIZ 2023067) from China; E, the male specimen (KIZ 2023066) from China; F, the female specimen (KIZ 2023070) from China.

As some discrepancies between the morphological characters of the specimens we collected from the type locality of *Amolopsminutus* and the original descriptions of this species, in order to verify whether the specimens we collected belong to *A.minutus*, we rely on the type specimen of this species. Re-examination of the type specimens verified that the specimens collected from the type locality *A.minutus* belong to *A.minutus*. In addition, morphological comparison between the type specimens of *A.ottorum* and the type and topotypic specimens of *A.minutus* showed that these two species could not be clearly distinguished. Combined with phylogenetic analysis, we confirmed that *A.ottorum* and *A.minutus* are conspecific and *A.ottorum* should be regarded as a junior synonym of *A.minutus*. Moreover, we found that *A.minutus* is also distributed in China and the colouration of this species in life was quite variable (Fig. 4), which extends the distribution range and diagnosis of this species.

Guanyinshan Provincial Nature Reserve is located in southern Yunnan Province of China, with relatively high altitudes (approximately 1640–2746 m) and well-preserved natural habitat. There have been few previous field surveys in this nature reserve, resulting in a serious underestimation of its species diversity. Some new species of plants and animals have been discovered in this nature reserve recently, such as *Primulaweimingii* Bin Yang & Y.H. Tan and *Pareasguanyinshanensis* Liu, Mo, Li, Li, Luo, Rao & Li, 2024 (Yang et al. 2023, Liu et al. 2024). The discovery of *Amolopsminutus* in this nature reserve further enriches its species diversity. More field surveys will likely reveal more new species or new records of amphibians from this nature reserve.

## Supplementary Material

XML Treatment for Amolops minutus

## Figures and Tables

**Table 1.** Sequences (16S) used for molecular analyses in this study.

Species	Voucher	Locality	Accession number
*Amolopsailao*	GXNU YU000001	Xinping, Yunnan, China	MN650751
*Amolopsailao*	GXNU YU000002	Xinping, Yunnan, China	MN650752
*Amolopsailao*	GXNU YU000003	Xinping, Yunnan, China	MN650753
*Amolopsailao*	GXNU YU000004	Xinping, Yunnan, China	MN650754
*Amolopsailao*	GXNU YU20160273	Xinping, Yunnan, China	MN650755
*Amolopsailao*	GXNU YU20160274	Xinping, Yunnan, China	MN650756
*Amolopsailao*	KIZ 2022041	Xinping, Yunnan, China	OP879227
*Amolopsdafangensis*	MT DF20230601002	Dafang, Guizhou, China	OR936315
*Amolopsdafangensis*	MT DF20230601001	Dafang, Guizhou, China	OR936314
*Amolopsdafangensis*	MT DF20230601003	Dafang, Guizhou, China	OR936316
*Amolopsdafangensis*	MT DF20230601004	Dafang, Guizhou, China	OR936317
*Amolopsdafangensis*	MT DF20230601005	Dafang, Guizhou, China	OR936318
*Amolopsgranulosus*	SYS a005315	Hongya, Sichuan, China	MK604850
*Amolopsgranulosus*	SYS a005316	Hongya, Sichuan, China	MK604851
*Amolopsgranulosus*	20130258	Hongya, Sichuan, China	MH922934
*Amolopsjinjiangensis*	SCUM 050435CHX	Deqing, Yunnan, China	EF453741
*Amolopsjinjiangensis*	CIB-XM6120	Deqing, Yunnan, China	MZ292455
*Amolopsjinjiangensis*	KIZ 047095	Chuxiong, Yunnan, China	MN953701
*Amolopslifanensis*	SYS a005378	Lixian, Sichuan, China	MK604870
*Amolopsloloensis*	SYS a005351	Zhaojue, Sichuan, China	MK573806
*Amolopsloloensis*	SYS a005346	Zhaojue, Sichuan, China	MK604854
*Amolopsloloensis*	SYS a005347	Zhaojue, Sichuan, China	MK604855
*Amolopsmantzorum*	SYS a005366	Baoxing, Sichuan, China	MK604862
*Amolopsmantzorum*	SYS a005362	Baoxing, Sichuan, China	MG991893
*Amolopsmantzorum*	SYS a005336	Hongya, Sichuan, China	MK573804
*Amolopsminutus*	IEBR A.5142	Tam Duong, Lai Chau, Vietnam	PQ346023
*Amolopsminutus*	IEBR A.6300	Tam Duong, Lai Chau, Vietnam	PQ346024
*Amolopsminutus*	IEBR 4342 (Holotype of *A.ottorum*)	Muong La, Son La, Vietnam	PQ346025
*Amolopsminutus*	TBU 06 (Paratype of *A.ottorum*)	Muong La, Son La, Vietnam	PQ346026
*Amolopsminutus*	KIZ 2023064	Yuanyang, Yunnan, China	PQ346027
*Amolopsminutus*	KIZ 2023065	Yuanyang, Yunnan, China	PQ346028
*Amolopsminutus*	KIZ 2023066	Yuanyang, Yunnan, China	PQ346029
*Amolopsminutus*	KIZ 2023067	Yuanyang, Yunnan, China	PQ346030
*Amolopsminutus*	KIZ 2023068	Yuanyang, Yunnan, China	PQ346031
*Amolopsminutus*	KIZ 2023069	Yuanyang, Yunnan, China	PQ346032
*Amolopsminutus*	KIZ 2023070	Yuanyang, Yunnan, China	PQ346033
*Amolopsminutus*	KIZ 2023102	Yuanyang, Yunnan, China	PQ346034
*Amolopssangzhiensis*	CSUFT 901	Sangzhi, Hunan, China	OQ079538
*Amolopssangzhiensis*	CSUFT 905	Sangzhi, Hunan, China	OQ079539
*Amolopssangzhiensis*	CSUFT 907	Sangzhi, Hunan, China	OQ079540
*Amolopsshuichengicus*	SYS a004956	Shuicheng, Guizhou, China	MK604845
*Amolopsshuichengicus*	SYS a004957	Shuicheng, Guizhou, China	MK604846
*Amolopstuberodepressus*	CIB-XM3125	Jingdong, Yunnan, China	KR559270
*Amolopstuberodepressus*	YU20160272	Xinping, Yunnan, China	MN650757
*Amolopstuberodepressus*	SYS a003931	Jingdong, Yunnan, China	MG991904
*Amolopsviridimaculatus*	SYS a003813	Mt. Gaoligong, Yunnan, China	MK604836

**Table 2.** Comparisons amongst the type specimens of *Amolopsminutus*, the topotypic specimens of *A.minutus*, the type specimens of *A.ottorum* and the specimens of *A.minutus* collected from China. Measurements in mm. Abbreviations defined in the text. Data for the type specimens of *A.minutus* and *A.ottorum* were from the original descriptions (Orlov and Ho 2007; Pham et al. 2019).

	Types of *Amolopsminutus*		Topotypes of *Amolopsminutus*		Types of *Amolopsottorum*	*Amolopsminutus* from China	
	Males (n = 8)	Females (n = 5)	Males (n = 8)	Females (n = 7)	Females (n = 2)	Males (n = 6)	Females (n = 3)
SVL	29.7–36.4	38.5–50.2	30.8–34.0	42.5–47.5	47.5–48.2	34.6–38.3	46.7–51.1
HL	11.0–12.9	12.5–15.7	10.8–12.0	14.3–16.0	15.1–16.0	12.2–13.6	16.0–17.1
HW	9.7–11.4	10.1–14.4	10.0–11.0	13.5–14.0	14.6–14.9	10.9–11.8	15.1–16.6
ED	4.3–5.0	5.4–6.4	4.7–5.0	5.5–6.0	5.7–5.8	4.4–4.7	5.5–5.6
TD	2.1–2.7	3.1–3.5	1.8–2.0	2.2–2.4	2.1	1.3–1.4	1.5–1.8
ESL	4.8–5.4	6.5–7.6	4.9–5.4	6.4–7.0	6.8–7.0	5.6–6.0	7.2–7.7
FLL	25.7–28.2	32.7–40.3	22.5–25.5	30.5–32.5	32.3–32.5	26.6–28.6	34.8–38.8
HLL	59.5–67.2	74.3–85.5	58.2–65.6	81.5–87.7	82.3–83.5	72.4–75.9	92.3–99.3
TL	17.7–20.3	22.3–25.6	17.4–20.4	24.8–27.0	27.2–27.8	21.6–22.7	27.0–29.1
FOT	25.7–29.4	31.2–36.0	25.0–28.6	34.8–37.2	37.4–38.0	30.5–32.2	39.2–42.6
HL/SVL	0.33–0.37	0.31–0.33	0.34–0.36	0.32–0.35	0.32–0.33	0.35–0.36	0.33–0.34
HW/SVL	0.28–0.33	0.26–0.31	0.30–0.33	0.29–0.32	0.31	0.30–0.32	0.32
ESL/SVL	0.14–0.17	0.15–0.17	0.15–0.16	0.15	0.14–0.15	0.15–0.16	0.14–0.16
ED/HL	0.36–0.44	0.36–0.44	0.41–0.44	0.36–0.39	0.36–0.38	0.34–0.36	0.33–0.34
TD/HL	0.19–0.23	0.20–0.27	0.17	0.14–0.16	0.13–0.14	0.10–0.11	0.09–0.11
FLL/SVL	0.77–0.87	0.71–0.85	0.70–0.75	0.68–0.72	0.67–0.68	0.72–0.77	0.73–0.76
HLL/SVL	1.79–2.00	1.70–1.93	1.87–1.94	1.81–1.92	1.71–1.76	1.98–2.09	1.85–2.00
TL/SVL	0.54–0.60	0.51–0.58	0.56–0.60	0.54–0.59	0.57–0.58	0.59–0.63	0.54–0.59
FOT/SVL	0.77–0.86	0.72–0.81	0.78–0.84	0.76–0.82	0.78–0.80	0.83–0.88	0.79–0.86

**Table 3.** Uncorrected pairwise genetic distances (%) estimated from 16S rRNA sequences.

	1	2	3	4	5	6	7	8	9	10	11	12
1 Types of *Amolopsottorum*												
2 Topotypes of *Amolopsminutus*	0.7											
3 *Amolopsminutus* from China	0.6	0.6										
4 *Amolopsailao*	1.2	1.8	1.6									
5 *Amolopsdafangensis*	2.1	1.9	1.8	1.7								
6 *Amolopsgranulosus*	2.2	2.8	2.5	2.7	1.8							
7 *Amolopsjinjiangensis*	1.6	1.6	1.4	2.1	1.1	2.1						
8 *Amolopslifanensis*	5.8	8.0	8.4	8.3	4.8	7.6	7.3					
9 *Amolopsloloensis*	2.0	2.1	1.8	2.3	1.5	1.9	1.1	7.6				
10 *Amolopsmantzorum*	2.0	2.2	1.7	1.9	2.2	2.5	1.9	8.5	2.0			
11 *Amolopssangzhiensis*	1.4	1.9	1.6	1.8	0.8	2.0	0.8	7.8	1.4	1.7		
12 *Amolopsshuichengicus*	1.6	2.8	2.7	2.9	1.7	2.7	1.9	7.9	2.4	3.0	1.8	
13 *Amolopstuberodepressus*	1.8	2.7	2.2	2.5	2.1	2.1	1.7	8.2	1.8	1.8	1.8	2.8

## References

[B11709041] Dever Jennifer A., Fuiten Allison M., Konu Özlen, Wilkinson Jeffery A. (2012). Cryptic torrent frogs of Myanmar: An examination of the *Amolopsmarmoratus* species complex with the resurrection of *Amolopsafghanus* and the identification of a new species. Copeia.

[B11709059] Fei Liang, Hu Shuqin, Ye Changyuan, Huang yongzhao (2009). Fauna Sinica. Amphibia Vol. 2 Anura.

[B11709076] Fei Liang, Ye Changyuan, Jiang Jianping (2012). Colored atlas of Chinese amphibians and their distributions.

[B11709093] Frost Darrel Amphibian species of the world: an online reference. Version 6.1.. http://research.amnh.org/herpetology/amphibia/index.html.

[B11709142] Hedges S (1994). Molecular evidence for the origin of birds. Proceedings of the National Academy of Sciences.

[B11709101] Jiang Ke, Ren Jionglong, Lyu Zhingtong, Wang Dan, Wang Zeng, Lv Ke, Wu Jiawei, Li Jiatang (2021). Taxonomic revision of *Amolopschunganensis* (Pope, 1929) (Amphibia: Anura) and description of a new species from southwestern China, with discussion on *Amolopsmonticola* group and assignment of species groups of the genus *Amolops*. Zoological Research.

[B11709151] Kalyaanamoorthy Subha, Minh Bui, Wong Thomas, von Haeseler Arndt, Jermiin Lars (2017). ModelFinder: fast model selection for accurate phylogenetic estimates. Nature Methods.

[B11709161] Katoh K., Standley D (2013). MAFFT Multiple Sequence Alignment Software Version 7: Improvements in Performance and Usability. Molecular Biology and Evolution.

[B11709170] Li Shize, Liu Jing, Ke Xiaocong, Cheng Gang, Wang Bin (2024). ﻿A new species of *Amolops* (Amphibia, Anura, Ranidae) from Guizhou Province, China. ZooKeys.

[B11709180] Liu Shuo, Mo Mingzhong, Li Mei, Li Biao, Luo Xiong, Rao Dingqi, Li Song (2024). Description of a new species of the *Pareashamptoni* complex from Yunnan, China, with confirmation of *P.hamptoni* sensu stricto in China (Squamata, Pareidae). Animals.

[B11709193] Mahony S, Nidup T, Streicher J, Teeling E, Kamei R (2022). A review of torrent frogs (*Amolops*: Ranidae) from Bhutan, the description of a new species, and reassessment of the taxonomic validity of some *A.viridimaculatus* group species aided by archival DNA sequences of century-old type specimens. The Herpetological Journal.

[B11709206] Matsui Masafumi, Shimada Tomohiko, Liu Wanzhao, Maryati Mohamed, Khonsue Wichase, Orlov Nikolai (2006). Phylogenetic relationships of Oriental torrent frogs in the genus *Amolops* and its allies (Amphibia, Anura, Ranidae). Molecular Phylogenetics and Evolution.

[B11709217] Nguyen Lam, Schmidt Heiko, von Haeseler Arndt, Minh Bui (2015). IQ-TREE: A fast and effective stochastic algorithm for estimating maximum-likelihood phylogenies. Molecular Biology and Evolution.

[B12040477] Orlov NL, Ho CT (2007). Two new species of cascade ranids of *Amolops* genus (Amphibia: Anura: Ranidae) from Lai Chau Province (northwest Vietnam). Russian Journal of Herpetology.

[B11709256] Pham Anh, Sung Nenh, Pham Cuong, Le Minh, Ziegler Thomas, Nguyen Truong (2019). A new species of *Amolops* (Anura: Ranidae) from Vietnam. The Raffles Bulletin of Zoology.

[B11709267] Ronquist Fredrik, Teslenko Maxim, van der Mark Paul, Ayres Daniel, Darling Aaron, Höhna Sebastian, Larget Bret, Liu Liang, Suchard Marc, Huelsenbeck John (2012). MrBayes 3.2: Efficient Bayesian phylogenetic inference and model choice across a large model space. Systematic Biology.

[B12066392] Simon Chris, Frati Francesco, Beckenbach Andrew, Crespi Bernie, Liu Hong, Flook Paul (1994). Evolution, Weighting, and Phylogenetic Utility of Mitochondrial Gene Sequences and a Compilation of Conserved Polymerase Chain Reaction Primers. Annals of the Entomological Society of America.

[B11709282] Tamura Koichiro, Stecher Glen, Kumar Sudhir (2021). MEGA11: Molecular Evolutionary Genetics Analysis Version 11. Molecular Biology and Evolution.

[B11709291] Tang Shangjing, Sun Tao, Liu Shuo, Luo Sangdi, Yu Guohua, Du Lina (2023). A new species of cascade frog (Anura: Ranidae: *Amolops*) from central Yunnan, China. Zoological Letters.

[B11709302] Wu Yun-He, Yan Fang, Stuart Bryan, Prendini Elizabeth, Suwannapoom Chatmongkon, Dahn Hollis, Zhang Bao-Lin, Cai Hong-Xia, Xu Yong-Biao, Jiang Ke, Chen Hong-Man, Lemmon Alan, Lemmon Emily, Raxworthy Christopher, Orlov Nikolai, Murphy Robert, Che Jing (2020). A combined approach of mitochondrial DNA and anchored nuclear phylogenomics sheds light on unrecognized diversity, phylogeny, and historical biogeography of the torrent frogs, genus *Amolops* (Anura: Ranidae). Molecular Phylogenetics and Evolution.

[B11710190] Wu Yun-He, Yu Zhong-Bin, Lu Chen-Qi, Zhang Yin-Peng, Dong Wen-Jie, Liu Xiao-Long, Kilunda Felista-Kasyoka, Xiong Yun, Jiang Yun-Fang, Ouyang Hong, Fu Zhong-Xiong, He Yun-Biao, Yuan Zhi-Yong, Che Jing (2024). A new species of the genus *Amolops* (Amphibia: Ranidae) and the first national record of *Amolopsvitreus* from China. Vertebrate Zoology.

[B11709333] Yang B, Zuo Y, Li M, Quan D, Wang L, Zhou S, He G, Tan Y (2023). *Primulaweimingii* (Primulaceae), a new species from Yunnan, China. Taiwania.

